# Autoantibodies to Peptidylarginine Deiminase 2 Are Associated With Less Severe Disease in Rheumatoid Arthritis

**DOI:** 10.3389/fimmu.2018.02696

**Published:** 2018-11-20

**Authors:** Erika Darrah, Jon T. Giles, Ryan L. Davis, Pooja Naik, Hong Wang, Maximilian F. Konig, Laura C. Cappelli, Clifton O. Bingham, Sonye K. Danoff, Felipe Andrade

**Affiliations:** ^1^Division of Rheumatology, The Johns Hopkins University School of Medicine, Baltimore, MD, United States; ^2^Division of Rheumatology, College of Physicians and Surgeons, Columbia University, New York, NY, United States; ^3^Division of Pulmonary and Critical Care Medicine, The Johns Hopkins University School of Medicine, Baltimore, MD, United States

**Keywords:** rheumatoid arthritis, peptidylarginine deiminase, autoantibodies, autoimmunity, disease activity, interstitial lung disease, Sharp score, shared epitope

## Abstract

**Objective:** Peptidylarginine deiminases (PAD) 2 and 4 are key enzymes in rheumatoid arthritis (RA) pathogenesis due to their ability to generate the protein targets of anti-citrullinated protein antibodies (ACPA). Anti-PAD4 antibodies that cross-react with PAD3 (anti-PAD3/4) have been identified and are associated with severe joint and lung disease. Here, we examined whether anti-PAD2 antibodies were present in patients with RA and defined their clinical significance.

**Patients and Methods:** A PAD2 ELISA was established to screen for anti-PAD2 IgG in sera from RA patients from a prospective observational cohort study (*n* = 184) and healthy controls (*n* = 100). RA patient characteristics were compared according to anti-PAD2 antibody status. Multivariable models were constructed to explore the independent associations of anti-PAD2 antibodies with clinical variables.

**Results:** Anti-PAD2 antibodies were found in 18.5% of RA patients and 3% of healthy controls (*p* < 0.001). Among RA patients, anti-PAD2 antibodies were not associated with traditional genetic or serologic RA risk factors, including HLA-DRβ1 shared epitope alleles, ACPA, rheumatoid factor (RF), or anti-PAD3/4 antibodies. In addition, antibodies to PAD2 were associated with fewer swollen joints, a lower prevalence of interstitial lung disease, and less progression of joint damage. In subset analyses in which patients were stratified by the baseline presence of ACPA/RF or anti-PAD3/4 antibodies, anti-PAD2 antibodies provided additional value in identifying patients with the least progressive joint disease.

**Conclusions:** Anti-PAD2 antibodies represent a novel serologic marker in RA that identifies a genetically and clinically unique subset of patients with less severe joint and lung disease.

## Introduction

Rheumatoid arthritis (RA) is a systemic autoimmune disease characterized by immune-mediated damage of synovial joints that affects ~1% of the population (1). There is marked heterogeneity in the clinical presentation, disease course, involvement of extra-articular organs, and response to therapy observed among individuals with RA, but the mechanisms driving this diversity are poorly understood. Anti-citrullinated protein antibodies (ACPAs), detected by the anti-cyclic citrullinated peptide (CCP) assay, are hallmark serologic features of patients with RA and serve as valuable diagnostic biomarkers (2). ACPAs are associated with specific *HLA-DR*β*1* alleles that confer genetic risk for RA development, collectively referred to as “shared epitope” (SE) alleles (3). Although, ACPA-positive patients with RA tend to have more severe disease on average than ACPA-negative individuals, the clinical heterogeneity in this group precludes the use of ACPAs as sole prognostic biomarkers (2). Precise markers that specifically identify clinically unique subgroups may reveal distinct underlying disease mechanisms with differences in prognosis and response to treatment.

The citrullinated protein targets of ACPAs are generated through the calcium-dependent deimination of arginine residues by the peptidylarginine deiminase (PAD) enzymes (4). There are five members of the PAD enzyme family (PAD1, PAD2, PAD3, PAD4, and PAD6), which display diverse tissue distribution. PAD6 is the only member without citrullination activity (5). PAD2 and PAD4 are implicated as central drivers of RA pathogenesis. Polymorphisms in the *padi2* and *padi4* genes are independently associated with RA development in Asian populations; PAD2 and PAD4 are observed in the tissue and fluid of inflamed RA joints; and both enzymes can generate citrullinated autoantigens (6–12). Interestingly, autoantibodies to PAD4 are present in ~35% of patients with RA and are associated with ACPAs and erosive joint disease (13–15). Moreover, a subgroup of anti-PAD4 antibodies that cross react with PAD3 (anti-PAD3/4 antibodies) is associated with the most severe and progressive joint disease and increased risk of interstitial lung disease (ILD) (16–19). Despite the appreciation that both PAD2 and PAD4 are important for RA pathogenesis, it is unknown whether PAD2 is also a target of the humoral response in RA. In this study, we sought to define the prevalence and clinical significance of anti-PAD2 antibodies in patients with RA.

## Patients and methods

### Human subjects

Sera from 100 healthy control volunteers and 184 RA patients from the Evaluation of Subclinical Cardiovascular Disease and Predictors of in RA (ESCAPE RA) cohort were screened for the presence of PAD2 autoantibodies by ELISA. All samples were obtained under informed written consent approved by the Johns Hopkins Institutional Review Board.

ESCAPE RA is a longitudinal cohort that has been extensively described previously (20, 21). Patients in this cohort met the American Rheumatism Association 1987 revised criteria for the classification of RA (22). Baseline demographic data and medication use were captured by questionnaire; anti-CCP, RF, and anti-PAD3/4 antibodies were measured as previously reported (16, 20, 21); and clinical features were assessed by clinical examination. Radiographs of the hands and feet, obtained at baseline and a follow-up visit occurring 39 ± 4 months after baseline, were scored according to the Sharp-van der Heijde method by an experienced reader blinded to clinical characteristics. The change in Sharp-van der Heijde score (SHS) between the two visits was calculated. The number of swollen joints was recorded at each study visit and the average mean swollen joint count (SJC) throughout the duration of the study was determined using an area-under-the-curve calculation. Participants underwent multidetector row computed tomography (MDCT) of the chest at the baseline visit, and the presence and extent of ILD was scored by an experienced pulmonary radiologist in a blinded fashion as previously described (21).

### Anti-CCP and HLA-DRβ1 typing

Anti-CCP antibodies and SE status were determined by anti-CCP2 ELISA and direct polymerase chain reaction sequencing of the HLA-DRβ1 gene, respectively, as previously described (20, 21). HLA-DRβ1^*^0101, 0102, 0401, 0404, 0405, 0408, 1001, 1402 were designated as SE alleles (3). HLA-DRβ1 alleles were also grouped according to the classification system suggested by Gourraud et al. in which S1 alleles were defined as HLA-DRβ1^*^0103, 0402, 1102, 1103, 1301, 1302, 1323, 15; S2 alleles were defined as HLA-DRβ1^*^0401 and 1303; S3D alleles were defined as HLA-DRβ1^*^1101, 1104, 12, 16; S3P alleles were defined as HLA-DRβ1^*^0101, 0102, 0404, 0405, 0408, 1001, 1402; and X alleles were defined as 03, 0403, 0407, 0411, 07, 08, 0901, 1401, 1404 (23).

### PAD2 protein purification

Recombinant human PAD2 protein was expressed from the pET28a vector, generating a fusion protein containing both N-terminal 6 × histidine and T7 tags. The protein was purified using a Ni-NTA agarose column according to manufacturer's instructions (Qiagen). Following PAD2 purification, the 6xHis tag was removed by cleavage with thrombin.

### Anti-PAD2 ELISA

High-binding EIA plates (Costar) were coated overnight with 200 ng/well of PAD2 in phosphate buffered saline pH 7.4 (PBS) or PBS alone. Plates were blocked with 3% non-fat milk. Patient sera were diluted 1:250 in 1% milk/PBS/0.05% tween-20 and assayed in duplicate. A known positive patient serum was serially diluted and included as a standard on each plate. Anti-PAD2 units were assigned with the highest standard representing 10 anti-PAD2 arbitrary units (AU). Anti-PAD2 antibody binding was detected using a horseradish peroxidase-conjugated anti-human IgG secondary antibody (Jackson Immunoresearch) diluted 1:7500 in 1% milk/PBS/0.05% Tween-20. SureBlue TMB peroxidase substrate (KPL) was added to visualize antibody binding and an equal volume of 1M hydrochloric acid was added to stop the colorimetric reaction, before determining the absorbance at 450 nm with a 560 nm reference using a Perkin Elmer Victor 3 plate reader. The standard curve formed from the serially diluted positive control sera was used to calculate anti-PAD2 AU based on the average absorbance values for each unknown serum sample using WorkOut software minus background binding to PBS-coated wells. The threshold for positivity was set at two standard deviations above the mean of the healthy control sera.

### Statistical analyses

The difference in the mean anti-PAD2 AU of the RA patients compared with healthy controls was determined using unpaired Student's *t*-test, and the difference in proportion of anti-PAD2 positives between the groups was determined using a 2 × 2 contingency table with Fisher's exact test. For analysis of demographic and clinical variables, RA patients were grouped according to the presence or absence of anti-PAD2 antibodies, and characteristics were compared using Intercooled STATA12. Student's *t*-tests were used for group-wise comparisons of normally distributed continuous variables; the Kruskal-Wallis test was used for group-wise comparisons of non-normally distributed variables; and Chi-squared or two-sided Fisher's exact tests, as appropriate, were used for group-wise comparisons of categorical variables. We explored the independent association of anti-PAD2 level with radiographic progression and the frequency of radiographic ILD using multivariable ordinal logistic regression, adjusting for covariates associated with the outcomes of interest and anti-PAD2 level at the *p* < 0.20 level in univariate modeling. A similar modeling strategy was used in the context of multivariable linear regression to explore the association of anti-PAD2 with baseline and average swollen joint counts. A two-tailed alpha = 0.05 was used throughout.

## Results

### PAD2 is a target of autoantibodies in patients with RA

**Figure 1:**
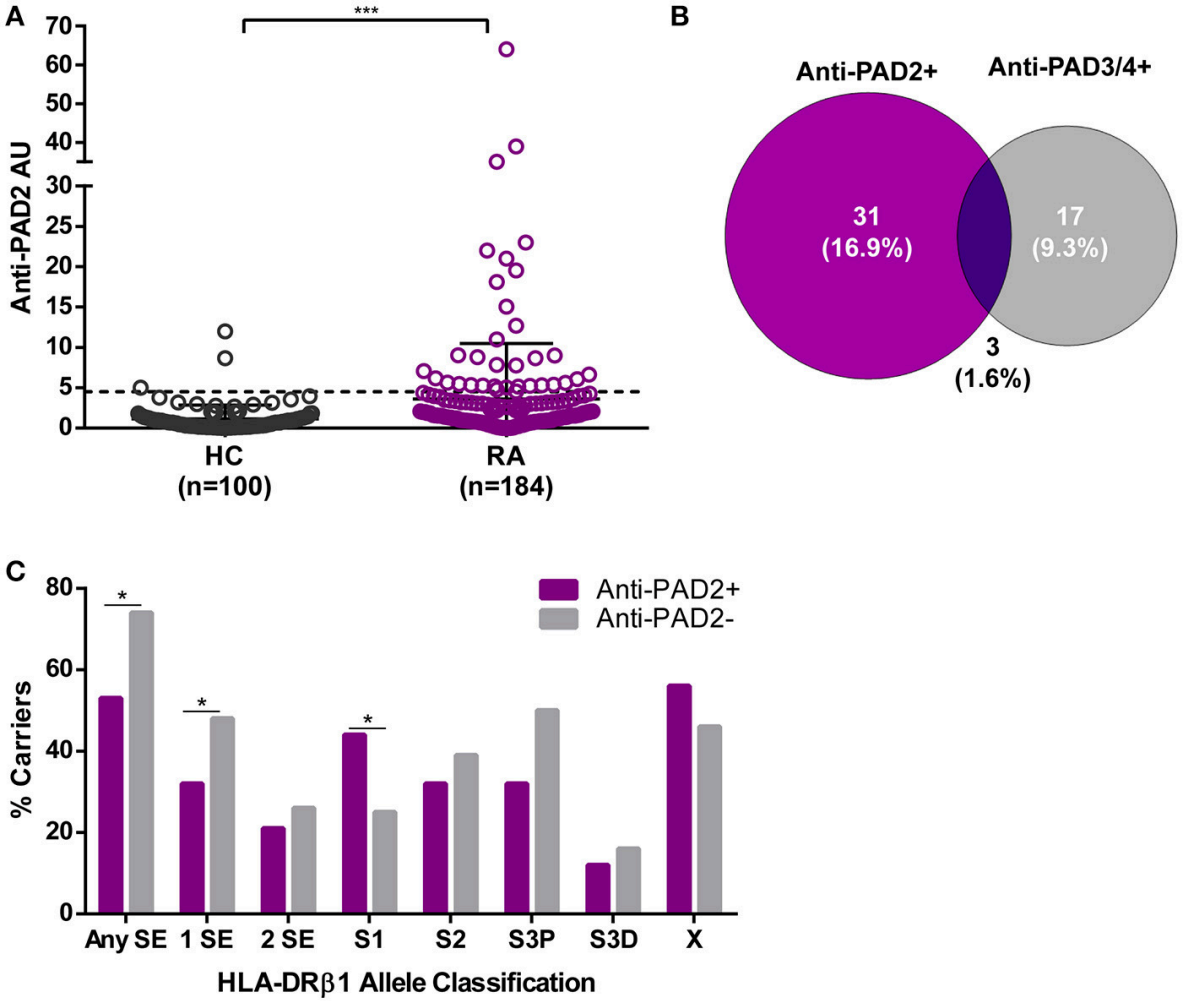
Anti-PAD2 antibodies are found in a serologically and genetically distinct subset of patients with RA. **(A)** Serum anti-PAD2 antibody arbitrary units (AU) are plotted for each RA patient and healthy control (HC). The cutoff for anti-PAD2 positivity is indicated (**—**) at 2-standard-deviations above the mean of the HC samples (Anti-PAD2 AU = 4.5). The median anti-PAD2 level in patients was compared to that of the healthy controls and a significant *p* < 0.001 is indicated (***). **(B)** A Venn diagram was created to illustrate the serologic overlap between anti-PAD2 and anti-PAD3/4 antibodies in the *n* = 183 patients for which both antibodies were measured. **(C)** The percentage of patients carrying different classes of HLA-DR alleles (% carriers) is shown on the y-axis according to anti-PAD2 antibody status, indicated on the x-axis. The % carriers for each HLA-DR class was compared between anti-PAD2 negative (gray bars) and anti-PAD2 positive (purple bars), and a *p* < 0.05 was indicated (*).

We established a PAD2 ELISA to define whether IgG antibodies to PAD2 were present in the serum of patients with RA. This assay was used to screen sera from 184 patients with established RA enrolled in the prospective observational ESCAPE-RA cohort and 100 healthy controls. The mean anti-PAD2 antibody level was significantly elevated in RA patients compared to healthy controls (*p* = 0.0005) (Figure 1A). Using a cutoff of two standard deviations above the mean anti-PAD2 antibody level in healthy sera, 18.5% (34/184) of RA patients and 3% (3/100) of healthy controls were positive for anti-PAD2 antibodies (*p* < 0.001).

### Anti-PAD2 antibodies identify a serologically and genetically distinct RA patient subset

To determine if anti-PAD2 antibodies were associated with specific serologic, genetic, or demographic characteristics within the RA population, patients were grouped according to their anti-PAD2 antibody status and variables collected at their baseline visit were compared (Table 1 and S1). Among patients with anti-PAD2 antibodies, 82% were female, a significantly higher proportion than present in the anti-PAD2 negative group (*p* = 0.003) (Table 1). Interestingly, there was no difference in the frequency of anti-CCP or RF according to anti-PAD2 antibody status (Table 1). In addition, there was little overlap between patients who had circulating antibodies to PAD2 and those who had anti-PAD3/4 antibodies (Table 1). Only 1.6% of patients generated both anti-PAD3/4 and anti-PAD2 antibodies (Figure 1B), suggesting that these autoantibodies may define two distinct subsets of patients with RA.

**Table 1:** Characteristics of ESCAPE RA patients according to anti-PAD2 antibody status.

**Patient characteristics**	**Anti-PAD2 negative *n* = 150**	**Anti-PAD2 positive *n* = 34**	***p*-value**
**DEMOGRAPHIC FEATURES**			
Age, years, mean ± SD	61 ± 8	63 ± 9	0.28
Male gender, *n* (%)	68 (45)	6 (18)	0.003
Caucasian, *n* (%)	131 (87)	28 (82)	0.44
Ever smoking, *n* (%)	88 (59)	21 (62)	0.74
Current smoking, *n* (%)	18 (12)	2 (6)	0.38
**SEROLOGIC FEATURES**			
RF positivity >40 units, *n* (%)	96 (64)	22 (65)	0.94
Anti-CCP positivity >20 units, *n* (%)	113 (76)	26 (76)	0.94
Anti-PAD3/4 positivity, *n* (%)	17(11)	3 (9)	0.66
**CLINICAL FEATURES**			
RA duration, years, median (IQR)	8 (4-17)	9.5 (7-19)	0.089
DAS28, median (IQR)	3.3 (2.5–4.0)	3.2 (2.5–3.9)	0.47
HAQ score (0–3), median (IQR)	0.75 (0.12–1.44)	1.0 (0.12–1.50)	0.19
CRP, mg/L, median (IQR)	2.9 (1.0–7.2)	3.3 (1.0–7.3)	0.91
Swollen joint count, median (IQR)	4 (2-8)	2 (1-6)	0.049
Tender joint count, median (IQR)	5 (2-14)	5 (2-12)	0.88
Nodules, *n* (%)	30 (21)^a^	3 (9)^b^	0.13
Any ILD, *n* (%)	50 (36)^c^	5 (18)^d^	0.060
Total SHS, median (IQR)	7(1-42)	12 (0–55)	0.91
Total erosion score, median (IQR)	3 (0–14)	3 (0–21)	0.83
Total JSN score, median (IQR)	5 (0–27)	8 (0–28)	0.69
Δ SHS (per year), median (IQR)	0.34 (0–2.1)^e^	0 (0–1.18)^f^	0.15
Any increase in SHS, *n* (%)	69 (58)^e^	13 (43)^f^	0.15
**CURRENT TREATMENT**			
Non-biologic DMARDs, *n* (%)	123 (83)	31 (91)	0.30
Biologic DMARDs, *n* (%)	64 (43)	19 (56)	0.17
Glucocorticoids, *n* (%)	60 (40)	13 (38)	0.85
Cumulative prednisone, g, median (IQR)	3.2 (0.5–8.7)	3.0 (0–11.7)	0.63

*SD, standard deviation; IQR, interquartile range; RF, rheumatoid factor; DAS, disease activity score; HAQ, health assessment questionnaire; CRP, C-reactive protein; SHS, Sharp van der Heijde score*.

a *n = 145*.

b *n = 34*.

c *n = 139*.

d *n = 28*.

e *n = 118*.

f *n = 30*.

The lack of association of anti-PAD2 antibodies with anti-CCP and anti-PAD3/4 antibodies, known serologies that associate with HLA-DRβ1 SE alleles (16, 17, 24, 25), suggested that anti-PAD2 may identify an immunogenetically distinct subset of RA patients. Indeed, individuals with anti-PAD2 antibodies were significantly less likely to have SE alleles compared with anti-PAD2 negative patients (53% vs. 74%, respectively) (Figure 1C). This effect was most strongly observed in patients who harbored only 1 SE allele (Figure 1C).

To identify HLA-DR alleles that may be enriched in patients with anti-PAD2 antibodies, an alternative classification strategy designated by Gourraud et al. was utilized (23, 26). Patients were stratified into five categories according to the presence of S1, S2, S3P, S3D, and X alleles (defined in Patients and Methods). These alleles are grouped based on the presence (S2 and S3P) or absence (S1, S3D, and X) of the classic SE motif, as well as, the identity of the amino acid present at position 71 of the peptide binding groove. S2 and S3P alleles have been reported to associate with severe RA and positivity for anti-CCP and RF, whereas S1 and S3D alleles were found to associate with more benign forms of RA and seronegativity. Using this strategy, anti-PAD2 antibodies were found to be significantly increased in patients harboring S1 alleles (alleles lacking the traditional SE motif) (Figure 1C).

### Anti-PAD2 antibodies identify patients with less severe baseline joint inflammation and fewer ILD features

**Figure 2:**
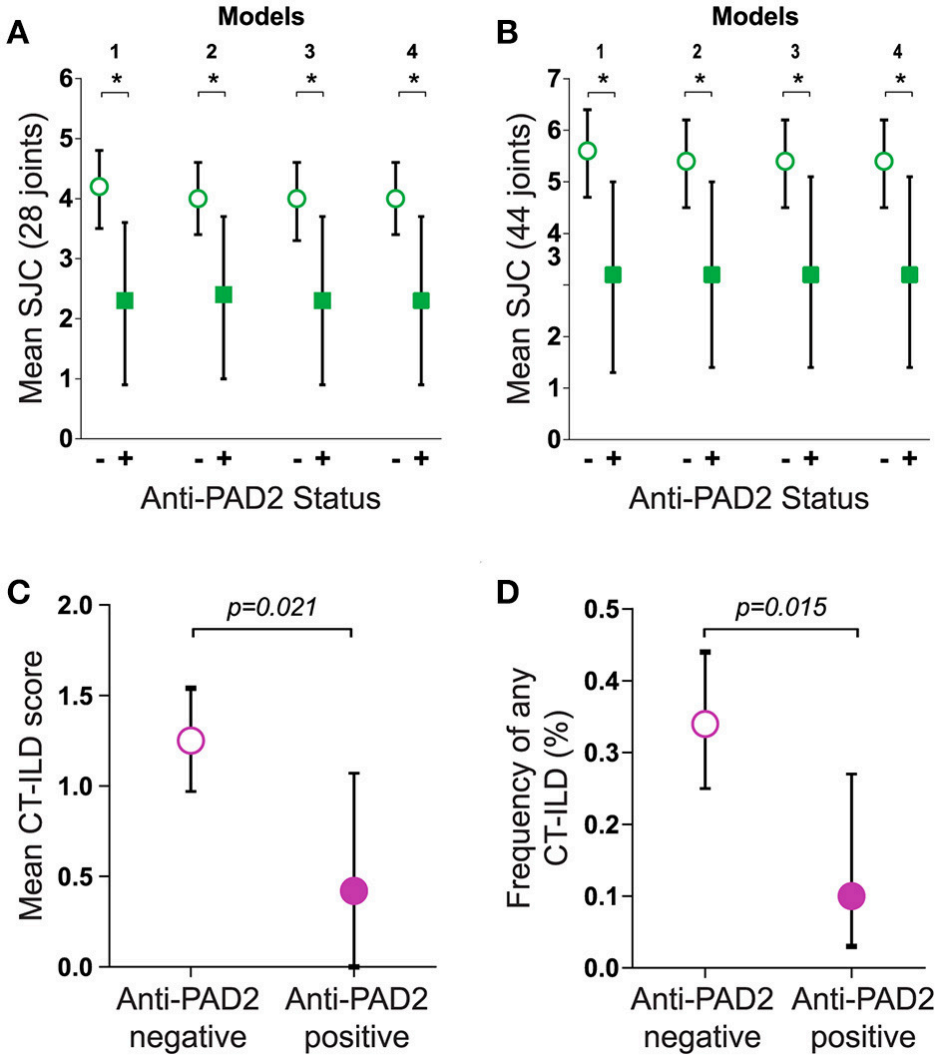
Anti-PAD2 antibodies are associated with fewer swollen joints and less ILD. Baseline mean SJC of **(A)** 28 or **(B)** 44 joints according to anti-PAD2 antibody status is shown. **(A,B)** Model 1 is unadjusted. Model 2 is adjusted for sex, BMI, RA duration, HAQ, biologic use. Model 3 is additionally adjusted for RF, anti-CCP, anti-PAD3/4, and SE. Model 4 is additionally adjusted for CRP. **(C)** The mean CT-ILD score, and **(D)** frequency of ILD adjusted for age, ever and current smoking, RF, anti-CCP, anti-PAD3/4, DAS28, and current biologic use is shown according to anti-PAD2 antibody status. **(A–D)** The average values, group 95% confidence intervals, and error bars are shown. A *p* < 0.05 was considered significant (*).

The finding that anti-PAD2 antibodies were not associated with traditional risk factors for severe disease (e.g., SE alleles and anti-CCP antibodies) suggested that they may identify a clinically unique RA patient subset. Analysis of baseline clinical variables revealed that patients with anti-PAD2 antibodies had a significantly lower median swollen joint count (SJC) at baseline, compared to those without anti-PAD2 (2 vs. 4 joints, respectively; *p* = 0.049) (Table 1). This was observed whether 28 or 44 joints were evaluated (Figures 2A,B). After adjusting for sex, body-mass index (BMI), disease duration, health assessment questionnaire (HAQ), biological DMARD use, known serologies, presence of SE alleles, and C-reactive protein (CRP) levels in reduced and fully adjusted models, anti-PAD2 antibodies remained independently associated with fewer swollen joints (*p* < 0.05, Figures 2A,B). The presence of ILD was also less prevalent in anti-PAD2 antibody positive vs. negative patients (18% vs. 36%, respectively; *p* = 0.06). It is important to note that the average ILD score among patients in this cohort was low, since patients were classified as having radiographic ILD if any features of ILD were seen on MDCT, irrespective of clinical symptoms (17). The lower frequency of ILD associated with anti-PAD2 was primarily driven by a difference in the presence of ground glass opacification (GGO) as the predominant radiographic ILD pattern. While 20 of the 131 anti-PAD2 negative patients (15%) had predominant GGO, 0% of the anti-PAD2 positive individuals (p = 0.026) had this radiographic finding. In contrast, 31 of the 131 patients (24%) without anti-PAD2 antibodies had reticulation, honeycombing, or traction bronchiectasis as their predominant ILD pattern, compared with 5 of the 27 (19%) patients with anti-PAD2 antibodies (p = 0.80). As such, the CT-ILD score was 66% lower in patients with anti-PAD2 antibodies compared to those who were anti-PAD2 negative (p = 0.021, Figure 2C). Even after adjusting for age, smoking history, known RA serologies, DAS28, and current biologic use, anti-PAD2 antibodies remained strongly and significantly associated with a lower frequency of ILD (_adj_OR = 0.24; *p* = 0.015) (Figure 2D).

### Anti-PAD2 antibodies are inversely associated with the progression of joint disease

**Figure 3:**
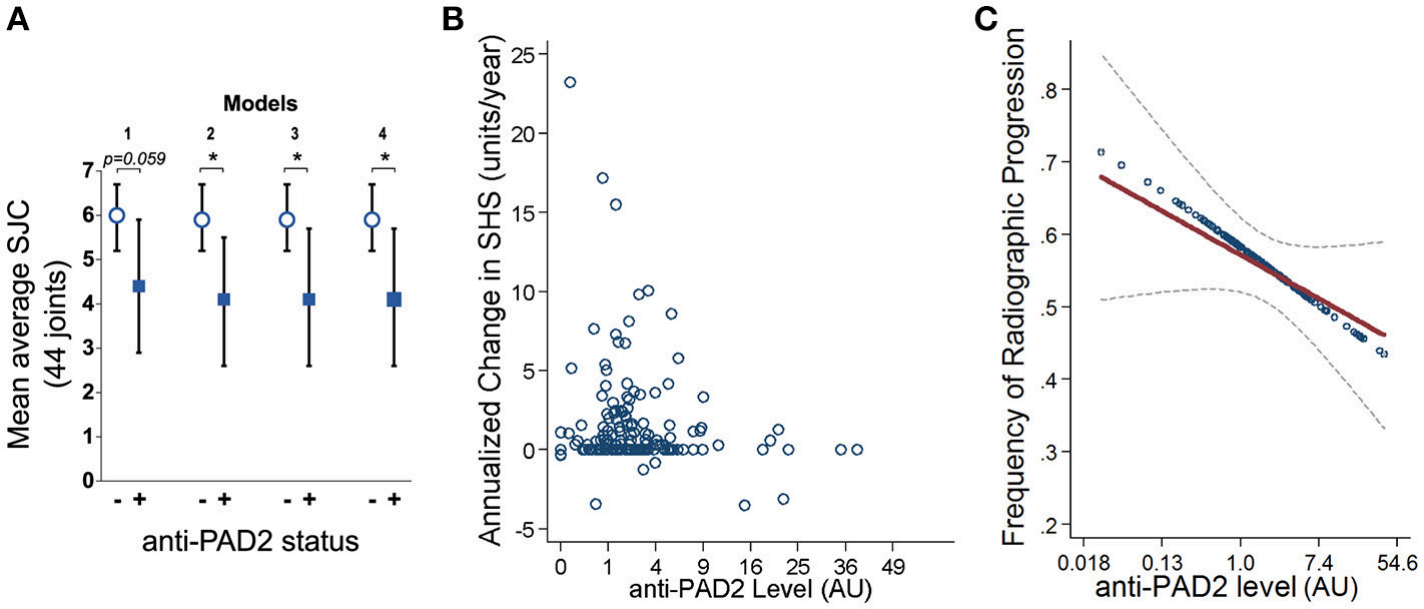
Anti-PAD2 antibodies are inversely associated with progressive joint damage. **(A)** Mean average SJC over all three visits is shown according to their anti-PAD2 antibody status. The models are adjusted for the co-variates indicated in Figures 2A,B. **(B)** Yearly change in SHS is plotted versus anti-PAD2 units for each patient. **(C)** Anti-PAD2 antibody level was plotted against the frequency of radiographic progression in unadjusted (blue circles) and adjusted (red line) models, and the least squares estimate of the association from multivariable linear regression with its associated 95% confidence interval (gray dotted line) is shown. A *p* < 0.05 was considered significant (*). AU, arbitrary units.

Since anti-PAD2 antibodies were independently associated with fewer swollen joints at baseline, they may also be independent markers of a less severe or less progressive arthritis phenotype. This hypothesis is supported by the finding that the average SJC, assessed at baseline and at two additional time points (with the final visit occurring an average of 39 ± 4 months after enrolment), was significantly lower in patients with anti-PAD2 antibodies, even in reduced and adjusted multivariable models (Figure 3A). Furthermore, the yearly change in SHS, a radiographic measure of joint damage, was negatively associated with anti-PAD2 antibody level. On average, each anti-PAD2 unit was associated with 0.08 SHS unit per year lower rate of radiographic progression (i.e., β = −0.08; *p* = 0.028) (Figure 3B). In a multivariable model adjusting for average CRP level, baseline SHS, and baseline adiponectin level, each log unit higher level of anti-PAD2 was associated with a 9% lower odds of radiographic joint disease progression (_adj_OR = 0.91; *p* = 0.016) (Figure 3C). Importantly, DMARDs use was not associated with protection from progression of radiographic joint damage in univariate models, suggesting that the association of anti-PAD2 antibodies with less progressive joint disease is independent of treatment.

### The clinical significance of anti-PAD2 antibodies in patients with RA

The lack of association of anti-PAD2 antibodies with anti-CCP or anti-PAD3/4 antibodies suggested that sub-setting patients based on these serologic markers may have prognostic value within the RA population. To address this, patients in the ESCAPE cohort were stratified according to anti-CCP or RF seropositivity, and the progression in radiographic joint damage as measured by annualized change in SHS or any change in SHS was determined according to anti-PAD2 antibody status (Table 2). In both groups, non-significant trends toward less progression of joint disease was observed in the anti-PAD2 positive subset with 15% fewer patients progressing compared to anti-PAD2 negative individuals. Patients were then stratified by the presence of baseline anti-PAD2 or anti-PAD3/4 antibodies only, as shown in Figure 2B, and radiographic progression was compared (Table 3). The number of patients who were positive for both antibodies (*n* = 3) was too small to include in the sub-analysis. As expected, patients who only had anti-PAD2 antibodies tended to have less progression in their joint disease than anti-PAD negative individuals, while patients who only had anti-PAD3/4 antibodies were 1.7-times more likely to have an increase in SHS over the course of the study compared to anti-PAD negative individuals. When the two antibody subsets were directly compared, the rate of progression was 2-fold higher in anti-PAD3/4 positive compared to anti-PAD2 positive individuals (*p* = 0.012) and the median annualized change in SHS was 0.8 (0.3–2.2) compared to 0 (0–1.2) (*p* = 0.046).

**Table 2:** Progression of joint damage according to anti-PAD2 and anti-CCP/RF status.

	**Anti-CCP and RF negative**			**Anti-CCP or RF positive**		
	**Anti-PAD2 negative (*****n*** = **33)**	**Anti-PAD2 positive (*****n*** = **7)**	***p*****-value**	**Anti-PAD2 negative (*****n*** = **86)**	**Anti-PAD2 positive (*****n*** = **23)**	***p*****-value**
Δ SvdH Score (per year), median (IQR)*	0.5 (0–2.1)	0 (0–1.2)	0.47	0.3 (0–1.7)	0 (0–1.3)	0.23
Any increase in SvdH score, *n* (%)*	19 (58)	3 (43)	0.68	50 (58)	10 (43)	0.21

* *Follow-up radiographs available in n = 149*.

**Table 3:** Progression of joint damage according to anti-PAD2 and anti-PAD3/4 status.

	**Group 1**	**Group 2**	**Group 3**		
	**Anti-PAD negative (*****n*** = **106)**	**Anti-PAD2 only (*****n*** = **28)**	**Anti-PAD3/4 only (*****n*** = **12)**	**Group 1 vs. 2** ***p*****-value**	**Group 1 vs. 3** ***p*****-value**	**Group 2 vs. 3** ***p*****-value**
Δ SvdH Score (per year), median (IQR)*	0.3 (0–2.0)	0 (0–1.2)	0.8 (0.3–2.2)	0.33	0.16	0.046
Any increase in SvdH score, *n* (%)*	58 (55)	13 (46)	11 (92)	0.43	0.014	0.012

* *Follow-up radiographs available in n = 146*.

## Discussion

Autoantibodies are useful biomarkers to understand disease mechanisms and distinguish clinical subsets within the autoimmune rheumatic diseases. Although major strides in autoantibody discovery have been made in RA, a sizable group of patients is seronegative and significant disease heterogeneity is observed within the seropositive population. This likely reflects the existence of unique disease subsets driven by distinct mechanisms. The precise definition of these disease subsets may allow correspondingly precise interventions in RA. The findings that anti-PAD2 antibodies are associated with less inflammatory and progressive joint disease and a lower frequency of CT-ILD, coupled to their lack of association with traditional RA-associated HLA alleles and serologies, set them apart from other RA autoantibodies described to date.

Although PAD2 and PAD4 enzymes are abundant in the RA joint (8, 9), it is intriguing that antibodies to both enzymes rarely co-exist in the same individual. It is unclear from this study whether stochastic factors or distinct pathogenic mechanisms are responsible for the generation of these autoantibodies. However, the differential association of these two antibodies with HLA-DR SE alleles and anti-CCP antibodies suggests that their development is driven by distinct processes which may also define unique clinical subsets within RA. Autoantibodies to the different PADs may have direct roles in defining the clinical course of disease. In this regard, it is possible that anti-PAD2 antibodies may be pathogenic but promote a less aggressive form of RA. Conversely, it is possible that the production of antibodies to PAD2 may help to attenuate the course of the disease, possibly by neutralizing or facilitating clearance of extracellular PAD2. The finding that the level of enzymatically active PAD2 in RA synovial fluid positively correlates with disease activity highlights the relevance of PAD2 in disease pathogenesis and how the production of neutralizing anti-PAD2 antibodies may be clinically beneficial (10, 27). Importantly, while further studies are necessary to define the role of anti-PAD2 antibodies in disease pathogenesis, the ability to identify patients who have a less severe prognosis is an important step toward the management of patients with RA, including minimizing risk of exposure to therapeutics with potentially dangerous side effects and lowering overall health care costs. Moreover, these studies suggest that in a subset of patients with RA, PAD2 may be playing an important modifiable role in disease pathogenesis.

The observational nature of the ESCAPE RA cohort and long average disease duration preclude us from answering questions related to treatment response outcomes and the prognostic potential of anti-PAD2 antibodies in pre- or early RA. Further studies with such cohorts and additional established RA cohorts are warranted to validate these findings and determine the breadth of clinical applications for this novel biomarker. Despite these outstanding questions, sub-analysis of RA patients by traditional RA serologies (i.e., anti-CCP and RF) and anti-PAD3/4 antibodies revealed a combinatorial value of including anti-PAD2 antibodies as prognostic biomarkers in patients with RA. It also revealed that 17.5% of the classically defined seronegative RA population (anti-CCP and/or RF negative) have anti-PAD2 antibodies, suggesting these may be clinically informative serologies in this poorly understood patient population. The discovery of anti-PAD2 antibodies that are not associated with traditional RA risk factors but are associated with fewer swollen joints, less radiographic ILD, and less progressive joint damage has important prognostic and mechanistic implications in RA.

## Ethics statement

This study was carried out in accordance with the recommendations of the Federal Policy on Protection of Human Subjects, U. S. Department of Health and Human Services with written informed consent from all subjects. The protocol was approved by the Johns Hopkins Medicine Institutional Review Board.

## Author contributions

ED and FA designed the study and guided data collection, interpretation, and analysis. JG provided clinical samples and data from patients in the ESCAPE RA cohort and performed statistical analysis. LC contributed to clinical data analysis. RD and MK developed the PAD2 ELISAs. RD and HW purified PAD2, performed ELISAs, and contributed to data analysis. PN contributed to data analysis. CB provided RA samples and aided in data interpretation. SD characterized and scored lung disease in patients from the ESCAPE RA cohort. All authors contributed to the preparation of this manuscript and approved the final version for publication.

### Conflict of interest statement

FA and ED received a research grant from Medimmune and ED received a research grant from Pfizer unrelated to this work. FA, ED, and JG are authors on issued Patent No. 8,975,033 entitled “Human Autoantibodies Specific for PAD3 which are Cross-reactive with PAD4 and their Use in the Diagnosis and Treatment of Rheumatoid Arthritis and Related Diseases”. ED previously served on the scientific advisory board for Padlock Therapeutics, Inc., and FA has served as a consultant for Bristol-Myers Squibb and Pfizer. ED and FA have a licensed agreement for the use of anti-PAD2 antibodies to identify patient subsets in rheumatoid arthritis. The remaining authors declare that the research was conducted in the absence of any commercial or financial relationships that could be construed as a potential conflict of interest.
